# Supplementary material for: Integration of plasma and CSF metabolomics with CSF proteomic reveals novel associations between lipid mediators and central nervous system vascular and energy metabolism
Source: Sci Rep. 2023 Aug 23;13:13752. doi: 10.1038/s41598-023-39737-8 (PMC10447532; doi:10.1038/s41598-023-39737-8)
Supplement: Supplementary file 1 — Supplementary Figure S1. [file 41598_2023_39737_MOESM1_ESM.pdf]

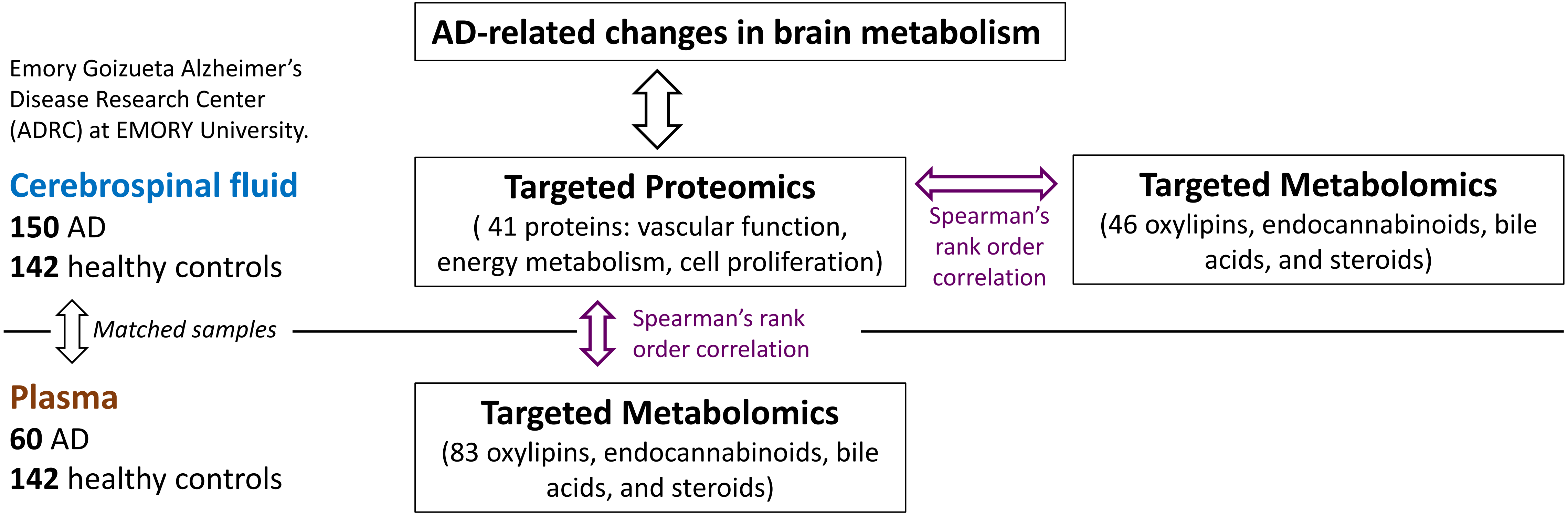

Figure S1. Overview of the study design. The cohort provided by the Emory Goizueta Alzheimer's Disease Research Center (ADRC) at EMORY University included 150 AD patients and 142 healthy controls. The cerebrospinal fluid (CSF) was available for all subjects. Plasma was available for all healthy controls and 60 AD patients. Available data included:

- 41 CSF proteins involved in the regulation of energy metabolism, vascular function, and cell proliferation. Those proteins are reflective of AD-related changes in the brain.
- 46 CSF lipid mediators, including oxylipins, endocannabinoids, PUFAs, bile acids, and steroids.
- 83 CSF lipid mediators, including oxylipins, endocannabinoids, PUFAs, bile acids, and steroids.
- The main statistical analysis included Spearman's rank-order correlation (purple color) between CSF proteomics and CSF metabolomics, and CSF proteomics and plasma metabolomics. Those analyses were stratified by the diagnosis group.
