# Supplementary material for: Integration of plasma and CSF metabolomics with CSF proteomic reveals novel associations between lipid mediators and central nervous system vascular and energy metabolism
Source: Sci Rep. 2023 Aug 23;13:13752. doi: 10.1038/s41598-023-39737-8 (PMC10447532; doi:10.1038/s41598-023-39737-8)
Supplement: Supplementary file 5 — Supplementary Figure S5. [file 41598_2023_39737_MOESM5_ESM.pdf]

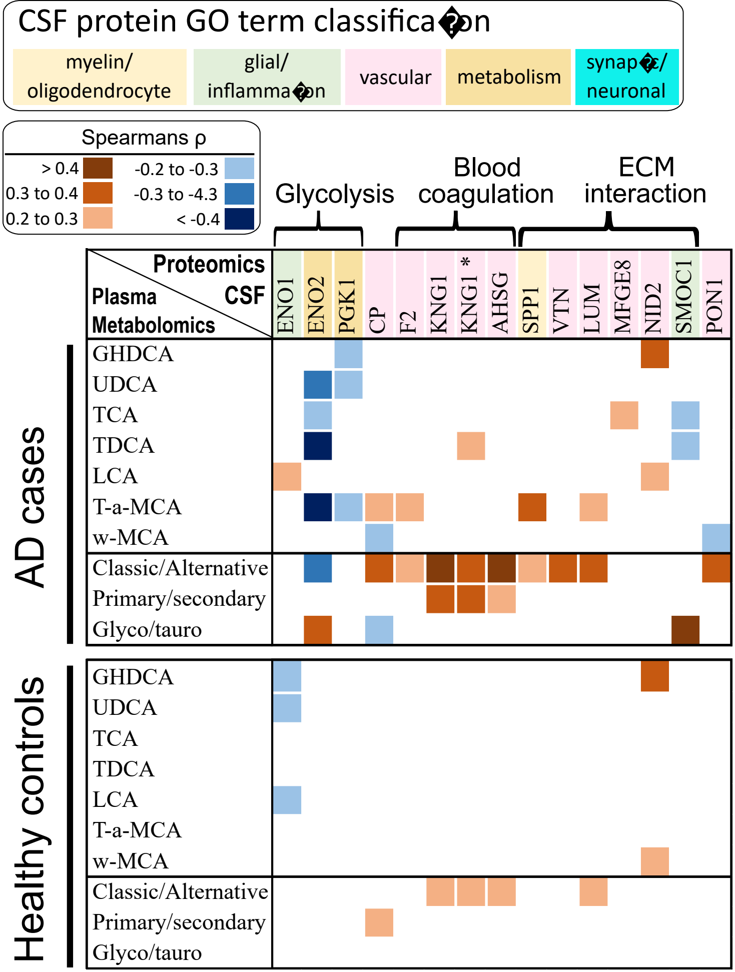

Figure S5. Spearman's ρ correlations between AD-implicated CSF proteins and plasma BAs. Only significant associations (p<0,05 and passing FDR corrected at q=0.2) are displayed. Proteins are colored according to Gene Ontology (GO) annotations. Additionally, proteins involved in glycolysis, blood coagulation and extracellular matrix (ECM) interaction are indicated. Spearman's ρ correlations between all analyzed CSF proteins and plasma metabolites are presented in the **Table S6.** N for AD cases = 60; healthy controls = 142. Two splicing variants are reported for KNG1 (P01042 and P01042-2, marked with asterisk). Classic/Alternative pathway is assessed using a ratio of conjugated BAs (TCA+GCA+TDCA+GDCA)/(GUDCA+TUDCA+GLCA+TLCA+TCDCA+GCDCA); Primary/Secondary ratio was assessed using DCA/CA ratio; Glyco/Tauro ratio was assessed using (GDCA+GLCA)/(TDCA+TLCA).
