# Supplementary material for: Integration of plasma and CSF metabolomics with CSF proteomic reveals novel associations between lipid mediators and central nervous system vascular and energy metabolism
Source: Sci Rep. 2023 Aug 23;13:13752. doi: 10.1038/s41598-023-39737-8 (PMC10447532; doi:10.1038/s41598-023-39737-8)
Supplement: Supplementary file 3 — Supplementary Figure S3. [file 41598_2023_39737_MOESM3_ESM.pdf]

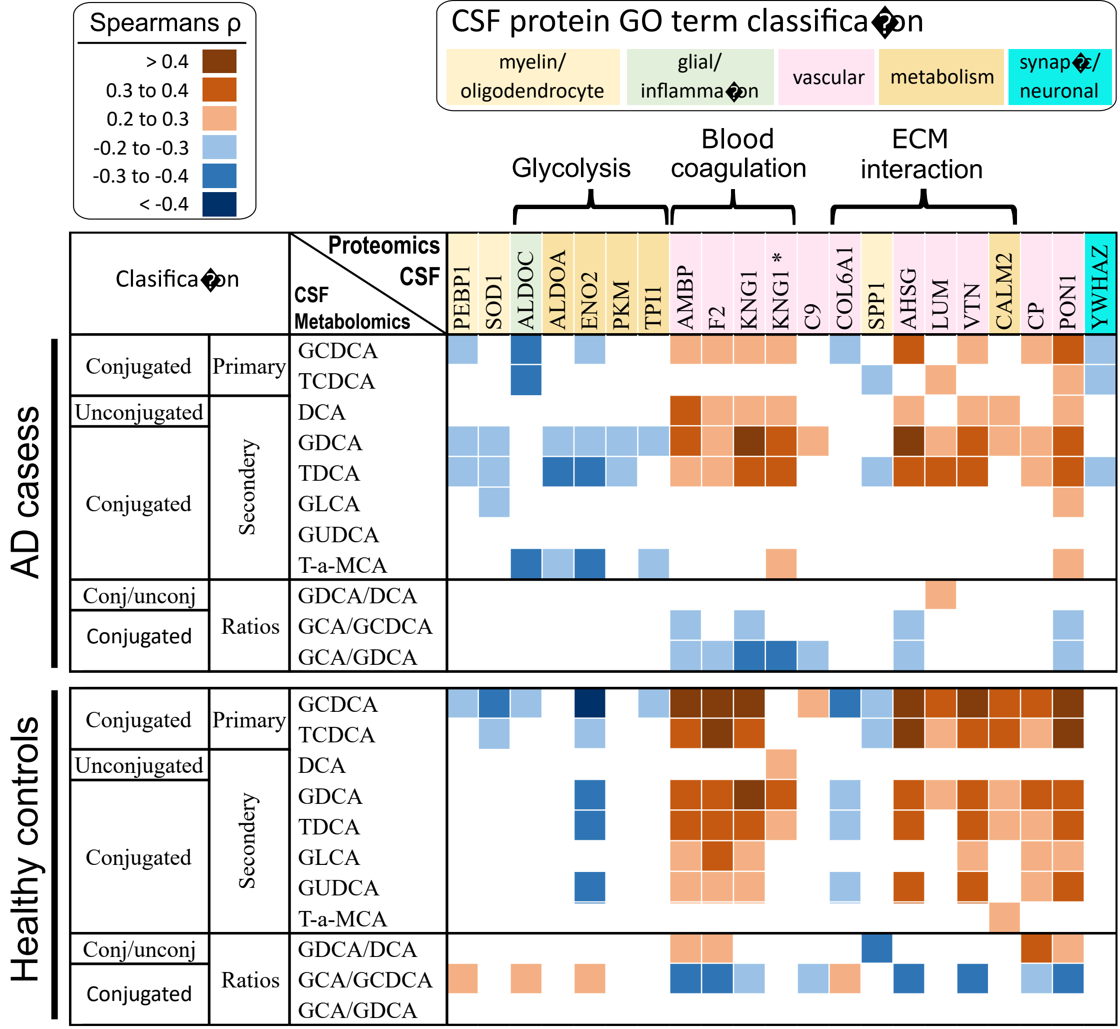

Figure S3. Spearman's *ρ* correlations between AD-implicated CSF proteins and CSF BAs. Only significant associations (p<0,05 and passing FDR corrected at q=0.2) are displayed. Associations are colored according to the Spearman's ρ, with positive associations on red and negative on blue scale. Proteins are colored according to Gene Ontology (GO) annotations. Additionally, proteins involved in glycolysis, blood coagulation and extracellular matrix (ECM) interaction are indicated. Spearman's *ρ* correlations between all analyzed CSF proteins and CSF metabolites are presented in **Table S3**. N for AD cases = 151; healthy controls = 142. Two splicing variants are reported for KNG1 (P01042 and P01042-2, marked with asterisk).
